# An Inquiry into the Anatomical Condition of the Alimentary Canal in Its Healthy State

**Published:** 1832

**Authors:** 


### WESTERN JOURNAL

OF THE

### MEDICAL AND PHYSICAL SCIENCES.

JULY, AUGUST AND SEPTEMBER, 1832.

### Essays and Cases.

Art. 1. *An Inquiry into the Anatomical Condition of the Alimentary Canal, in its Healthy State.* Extracted from the Pathological Anatomy of Professor Andral.

Editorial Note. We propose in due time, to lay before our readers, a full analysis of the comprehensive and deeply interesting Treatise of M. Andral on Pathological Anatomy, just republished and beginning to circulate in the United States. Meanwhile we are constrained to extract the entire chapter, on the appearance of the stomach and intestines after death from violence, or from diseases seated in other parts of the body. It is notorious, that very few physicians, in this country, are well acquainted with the appearances which may be presented by the gastro-enteric mucous membrane in its healthy condition; and none who are not, can be prepared to appreciate or report its morbid phenomena. We would especially commend this chapter to the attention of the profession, at a moment when they are about to grapple with a mortal epidemic, the fury of which seems to be in a great degree spent upon that membrane. By careful meditation upon the results of the numerous observations of which M. Andral is either the author or the historian, they will be better prepared to benefit themselves and the profession by their *post-mortem* examinations of those who may die of Cholera. In fact, it is indispensable, that they who would recognize and describe a morbid structure, should have a clear and vivid idea of its *healthy* condition. For want of this knowledge, many autopsic reports have undoubtedly abounded in mistakes, of which those who made them were entirely unconscious. A diligent study of the chapter we are about to present, must obviate much of this error; and give increased precision and value to our dissections. D.

*"Of the alimentary canal in the healthy state.*

"There has been hitherto so little agreement on the subject of the natural appearance of the stomach and intestines, that I consider it indispensable to determine accurately what is the anatomical condition of the alimentary canal in the healthy state. Perhaps one circumstance which has long been an obstacle to the ascertainment of this point, is the great frequency of grstro-intestinal alterations. As there are very few subjects in which some of these are not met with, anatomists had become accustomed to consider them as belonging to the natural state of the parts; and they seemed the more warranted in doing so, as, until very lately, the symptoms produced by these alterations were either totally unknown or ill understood.

If we examine the internal surface of the stomach or intestines in a living animal, that is not struggling, and whose circulation is not disturbed, we find it of a red tint somewhat deeper than that of the mucous membrane of the cheek in a healthy man. If we examine the same animal after death, we find that this red tint has disappeared, and that the surface is now uniformly pale, or, at most, very slightly rose-colored. In order that the experiment should afford these results, the animal must be deprived of life in such a manner as not to lose too much blood, on the one hand, as the natural paleness of the intestine would then be increased; or to die in a state of asphyxia, on the other, as the mucous membrane would then be mechanically injected; which, though not a morbid, would not yet be the natural state.

I think we may conclude from this experiment that, after death, the mucous membrane of the stomach and intestines tends to lose its color like the skin.

There have been frequent opportunities of examining bodies in cases of accidental death, where the individual was apparently in the enjoyment of perfect health a few minutes previously. In most of these cases, also, the alimentary canal has been found free from any red tint.

Sometimes, however, different degrees of injection have been observed on the internal surface of the stomach or intestines, as well in animals supposed to be sound, that were sacrificed to physiological experiments, as in men in cases of accidental death. To this it may be answered in the first place, that if the alimentary canal had been found oftener without any redness under the same circumstances, it is very probable that in the cases where the redness was observed, it arose from disease. But, besides, the appearance of the parts that were found injected should have been described with more care and precision; and a detailed account should have been given of the kind of death the animals suffered, and of the space of time that had elapsed between the accident and death in the other cases.

There are, in fact, certain circumstances under the influence of which the alimentary canal, though free from disease, may yet present various degrees of red coloration in the dead body. Of these circumstances, some may have operated a certain period before death, others only during the last moments, and lastly, others either soon or at some length of time after the cessation of life.

Of the causes which operate before death, some are physiological and others pathological. Thus, it is an undoubted fact, that, during the process of chymification, the internal surface of the stomach acquires a considerable degree of redness, as well as that the small intestine does the same while the separation of the chyle is taking place in it: any one may convince himself of the truth of these assertions by examining living animals. But, beside, it has been ascertained by observation that this redness that is produced by digestion continues after death; so that on opening the body of any individual that has died while chymification or chylification was going on within him, we shall find those portions of the alimentary canal in which the process had been taking place of an unusually high color.

The pathological causes are all such as act by presenting some obstacle to the free return of the venous blood from the gastro-intestinal parietes to the right cavities of the heart. There happens then to the mucous membrane of the alimentary canal what happens to the skin in persons who die of asphyxia: in such cases, we observe the cutaneous surface long before death acquiring a constantly an increasing color from the venous blood; now, what takes place in the skin must also take place in the intestine. We may assure ourselves directly of this by examining a coil of intestine in an animal who is slowly suffocating, when we shall find that, as the respiration becomes more difficult, the coil assumes a more intense and uniform red hue. Lastly, if, as Boerhaave did long ago, we prevent by a ligature the circulation of the blood in the trunk of the vena portæ, we shall observe the whole of the internal surface of the intestines assuming a fine red tinge, which is compared by Morgagni to the color of cochineal; and sometimes, even, blood transudes, through the parietes of the distended vessels, and fills the intestine. These facts being known, it is only drawing the conclusion from them to establish that, every time the blood cannot return freely from the capillaries of the intestinal mucous membrane to the venous trunks, that membrane will continue colored after death. Hence arise the various shades it presents in case of strangulated hernias, for instance, or of obstructions of the liver, of tumors situated on the course of the principal divisions of the vena portæ, of obliteration of the vein itself by old coagula, and, lastly, of organic affections of the heart. If, however, there was but little blood in the body, whether through defect or sanguification, or in consequence of recent copious bleedings, a considerable obstacle to the venous circulation, would produce a less intense coloration of the alimentary canal, than that which would arise from a slighter obstacle existing in a person whose vessels contained a great deal of blood a short time before death.

The red coloration of the gastro-intestinal parietes in consequence of some mechanical obstacle to the venous circulation, presents various degrees of intensity. In the lowest of these, the sub-mucous cellular tissue alone is colored, but not in its capillary network; it is traversed in various directions by bluish veins of pretty large calibre, which cease to be injected on arriving at the mucous membrane, while their other extremities are continuous with the mesenteric veins, which are themselves equally gorged with blood. In a higher degree of injection, depending quite as much on mechanical causes as the preceeding, the mucous membrane itself begins to assume a tinge, and, according to the size, number, and relative situation of the injected vessels perceptible to the naked eye, it exhibits either simple branches, separated by large colorless intervals, or ramifications of greater or less extent, produced by the injection of the smaller vessels, or lastly, a redness considerable enough to produce a complete opacity of the parietes where ever it exists. According as these various shades of coloring are extended or circumscribed, the result will be either a diffused redness of the intestine without any precise limits, or else streaks, stripes, patches, or mere points. In fact, there is not one of these appearances that may not be produced by a simple injection from a hyperæmia either mechanical or passive; and he would be strangely mistaken who should imagine that the dotted redness, for instance, more necessarily announces an active hyperæmia than does the simple congestion of some of the submucous veins. In these different cases, on attentively examining the injected parts, we may perceive that the injected vessels are directly continuous with the great veins subjacent to the mucous membrane, just as these latter are continuous with the mesenteric.

If the obstacle to the return of the blood from the intestines to the heart is still more considerable, or, if, what comes to the same thing, the obstacle not being increased, there is an increase of blood in the vessels, that fluid escapes from them, and becomes effused, either into the submucous cellular tissue, where it forms ecchymoses, or into the cavity of the intestine itself, where it communicates a reddish tint to the bile, mucous, or other matters that happen to be contained in it. The facility with which a liquid or gaseous injection may be made to penetrate into the intestinal cavity when driven into the mesenteric veins from the trunks towards their branches, explains how, under the influence of a considerable congestion of the same veins, a part of the blood contained must have a tendency to escape into the interior of the alimentary canal.

Thus, on summing up all that we have learned both from simple physiological reasoning, experiments on animals, and the examination of dead bodies, we are led to conclude, that the gastro-intestinal mucous membrane may be indifferently white or red, without either of these colors necessarily indicating that the membrane had been in a morbid state; it is either white or red, of various shades, according as there has existed before death some one of the conditions, mechanical, organic, or vital, which we have endeavored to explain. Now, as those which produce the red coloration exist the most frequently, it follows that, in the dead body, we should more frequently find the alimentary canal injected than colorless. But that is not all: after life has ceased, new causes arise which tend to produce new modifications in the color of the intestines, and to inject some parts of it much more strongly than they were at the moment of death. The causes of redness produced after death may be reduced to two principal ones: one, the weight of the blood, and the other, its transudation through the parietes of its vessels.

The first of these causes begins to act immediately after death. The reality of its existence has been fully proved by MM. Trosseau and Rigot, in a memoir abounding in interesting facts and ingenious views.

If we fix to a nail the two united extremities of a coil of intestine supplied by veins gorged with blood, we shall perceive, after a short time, the most dependent part of the coil constituting the middle of the convexity of the curve thus formed, assuming a very marked red color, while the less dependent parts become paler and paler, and the mesenteric veins get rid of the great quantity of blood they contained in the vessels of the intestinal parietes, provided it be liquid, changes its place after death, and tends to accumulate in those parts of them where it is attracted by the laws of gravitation. Now, if this takes place in a coil of intestine separated from the body, it must occur equally in the interior of the abdomen, where the same physical law must also act. To settle this point, MM. Trosseau and Rigot began by placing the bodies in such a situation that the influence of gravitation should be beyond all doubt. Thus, upon leaving in a vertical position for several hours, the bodies of some dogs that had been previously strangled, they ascertained that, in those parts of the intestines that had been thereby rendered very dependent, the mucous membrane was of a lively red, its villi were strongly injected, blood was effused into the interior, where it tinged the bile and the mucus, and there were ecchymoses in the submucous cellular tissue. In other dogs, which had likewise been strangled, but were then placed on the belly, the portions of intestines which were more dependent in this new position were most highly colored. In horses killed by cutting the spinal cord, and placed afterwards on their back, but yet so that the bodies were sometimes more inclined to the right, and sometimes to the left, they found in like manner that the coils which were the most strongly injected, nay, the only ones which were so at all, were those in which the law of gravitation should have exerted its greatest influence in these various positions. They also remarked in the course of their experiments, that those which were shrunk and contracted, so that their vessels were doubled on themselves, were not injected, although placed in a dependent position. In the horse, on the contrary, the superior portions of intestine, *which were remarkable for their great diameter*, were found to have their under side injected frequently enough. If, on opening the body of an animal which has just died, we fix certain portions of the alimentary canal in a dependent position, and examine them again some time afterwards, we find them injected, though they were not so at the time of the first examination. M. Trosseau tells us he opened, six hours after death, the body of a man who died of a typhus fever: on examining some dependent parts of the ileum, they appeared to have a slight red tint, while the superior parts were, on the contrary, colorless: the intestines were then replaced. Next morning, on examining those dependent coils which had not been opened the preceding day, as well as those which had been partially so, he found them full of mucus colored by the blood, which had imparted a violet tint to their internal membrane. These facts fully confirm my own observations. For this long time back, while examining the bodies opened at *La Charite*, I have constantly been struck with the circumstance that those coils of the small intestine which are more dependent than the rest, those, for instance, which are sometimes found sunk in the hollow of the pelvis, are also more strongly injected. It becomes a question whether this coloration from hypostasis can occur in the small intestine only; it certainly can occur with more facility there than elsewhere, by reason of its disposition, and of that of the vessels distributed to it. I am, however, strongly inclined to think that, in certain cases, the redness observed on the great extremity of the stomach, and on its whole posterior surface in general, (that being inferior in the subject,) results in like manner from this accumulation of blood by hypostasis. I am the more disposed to this opinion, from finding it mentioned in my notes that, in a case where a body had been laid upon the abdomen a short time after death, preparatorily to opening the spinal canal, and remained several hours in that position, the anterior part of the stomach was injected, and dotted with red, while the posterior part was pale. At the time I imagined it to have been caused by gastritis; but I should not be apt to think so now.

The redness of the intestinal parietes that is produced, wholly after death, by injection from hypostasis, the reality of which I have just now proved, presents various degrees or shades, like the redness from congestion, either mechanical or passive, that had been previously under consideration. Thus, we may find the villi highly colored, and even blood effused into the interior of the intestinal canal. This, however, very seldom happens, except in experiments on animals that are strangled, and kept in the vertical position for several hours after death. In such cases, in fact, every thing is most favorably disposed for the blood's being drawn in the greatest possible quantity to where it is attracted by the law of gravitation. Nothing similar has ever been observed in the horses killed by pithing, or by knocking on the head; and, in the human subject, the determination of the blood towards the most dependent parts of the alimentary canal, most commonly produces in it only an injection more or less strong of the mucous membrane or of the subjacent cellular tissue; which may produce either a diffused tint with an appearance of ramifications, or circumscribed blushes in form of points, spots, streaks, &c.

Injection from hypostasis begins to take place immediately after death, acquires its highest degree at the end of some hours, and ceases to be continued as soon as the blood, having cooled, begins to coagulate. Hence it follows, that in subjects whose temperature is long kept up either naturally or artificially, and in which the blood continues fluid, the injection of the intestines from hypostasis, will be much more decided than under the opposite circumstances. It will also be more considerable when, after acute diseases, a great deal of blood still remains in the system; and when, in consequence of a slow death, or of obstacles to the circulation, the intestinal veins were gorged with blood at the moment of the cessation of life.

As soon as a certain space of time has elapsed after death, a new cause of coloration begins to act: as soon as putrefaction begins to seize upon the body, the blood contained in the vessels, both large and small, of the gastro-intestinal parietes exudes through the membranes of those vessels, and is effused in variable quantities into the surrounding tissues, especially into the submucous cellular tissue. On this extravasation of the blood depend, for instance, the red spots almost always observed in the stomach along the veins of its great extremity,when the body is opened more than six and thirty or forty hours after death. These spots thus assembled along the course of the vessels are sometimes isolated, and sometimes grouped together and running into one another, and in this manner mark the surface of the stomach with streaks and bands of various figures. If after having observed the stomach in this condition, we leave it, and examine it again in a latter period, we find that the redness has increased, and that, moreover, it appears in a new form: it no longer exists solely along the vessels, but the whole surface of the stomach presents a tinge which has a constantly increasing tendency to become uniform; and a period at last arrives, when all the membranes, having become soaked with blood, are equally red; they may then have a tint almost similar to that which we observe on the internal surface of the arteries when stained by the contained blood. This kind of redness formed after death cannot, however, proceed to such a high degree, unless in cases where a certain quantity of blood existed in the vessels of the stomach at the moment of death; and as, from the effects of gravitation, that fluid accumulates towards the great extremity of the stomach in particular, it follows that it is there we should see the redness from transudation most strongly marked. It would be useless to attempt to fix precisely the period at which this transudation should commence; for, in order to do that, we should fix precisely the period at which putrefaction commences. Now, that period is very variable, as it depends, 1. on certain conditions relative to the body itself; such as the kind of death, the nature of the disease that produced it, &c.; and, 2. on certain external circumstances, especially on the thermometrical and hygrometrical states of the place in which the body is. Accordingly, when, in summer time, we open bodies that have been kept, since death, in warm beds, and in rooms of a temperature at least as high as that of the external air, it is usual to find, so soon as after four and twenty hours, very evident marks of transudation in the alimentary canal: in such cases, for instance, I have often found all the membranes of the great extremity of the stomach of a uniform red tinge. Under similar circumstances the coloring matter of the blood may likewise transude, spread over the internal surface of the canal, and mix with the fluids contained. I have ascertained this to be the case in most of the bodies I had occasion to examine in the very warm summers of 1825 and 1826.

The spleen, also, may suffer some blood to transude through its parietes; and this may then soak into the adjacent portion of the stomach and impart its color to it. This kind of coloration, however, is less frequent than that which results from the transudation of the blood through the parietes of the gastric vessels. One of the circumstances that must have some effect in producing a variation in this cause, is, undoubtedly, the difference of the states in which the blood may be that is contained in the spleen; as the more liquid it is, the more readily must it soak through the enveloping membrane of that organ.

Lastly, even at the time we are engaged in examining the internal surface of the alimentary canal in the dead body, we may often produce in its mucous membrane, by scraping it with the back of a scalpel, a redness which not only did not exist during life, but which was not even apparent before the scraping. The effect of this operation is to squeeze into the finest vessels of the mucous membrane, and into a single point in those, the blood which being before that dispersed in the mass of the surrounding vessels, was much less apparent: extravasation, even, may be produced in this manner. It is to be observed that this artificial redness can be produced only when there previously exists a certain quantity of blood in the mucous membrane or below it: It most commonly assumes a dotted form.

When we plunge a bladder filled with blood into different gases, the blood becomes singularly altered in color. It follows that whenever these same gases are developed in the intestines, they must also affect the blood similarly through the parietes of its vessels. In some cases that may occur a very short time after death; but these gases being in general produced only by putrefaction, it is not until that process is tolerably advanced, that the gases resulting from it can modify the color of the blood, and change into brown or green, &c., the red tint which existed during life, or was formed after death by hypostasis or imbibition.

The bile that is found in the alimentary canal after death, in more or less considerable quantities, most commonly lines its internal surface without tinging it; but sometimes the yellow matter soaks into the mucous membrane, combines intimately with it, and produces a yellow tinge, which cannot be removed by washing. This may exist only in isolated spots, or affect a great extent uniformly; and it is often found very strongly marked in the stomach, where bile does not naturally occur More than once, for instance, I have found the whole internal surface of the right half of that viscus stained uniformly of a fine ochre color. I am inclined to think that this may be owing to the presence of an acid in the stomach, which must have a tendency to separate from the bile its yellow matter, which in this free state is more readily imbibed by, and combined with, the adjacent tissues. It is thus that some explain the yellow tinge observed on the mucous membrane of the duodenum in certain cases of poisoning by sulphuric acid.

To sum up; the gastro-intestinal mucous membrane is not of one constant and invariable color in the healthy state. It is perfectly white only in a very small number of cases, which I have mentioned. Besides these, it offers, without ceasing to be sound, different degrees of coloring, depending, 1. on the passive hyperæmia which has always a tendency to take place in the last moments of life in the parts abounding in capillaries; 2. on mechanical obstacles to the venous circulation formed at a longer or shorter period before death; 3. on the hypostatic accumulation of blood towards the dependent parts; 4. on the transudation of blood through its vessels; 5. on another kind of transudation which may take place, in some cases at least, through the capsule of the spleen; 6. on the presence of different gases in the alimentary canal at the moment of death; 7. on the developement of other gases, at a longer or shorter period after death, when putrefaction takes place; 8. on the combination of the yellow matter of the bile with different parts of the gastro-intestinal mucous membrane; 9. and lastly, on the accidental introduction into the alimentary canal of different coloring principles that may stain its internal surface, and thus produce a color more or less perfectly resembling the result of a morbid state.

Of the colors produced by these different causes, some cannot be in any way confounded with that resulting from inflammation; others differ from it only by characters which are often but feebly marked; and, lastly, others, especially those mentioned under the heads 1 and 3, as also some varieties of those under the heads 2 and 4, exactly resemble the color that would result in the alimentary canal from the irritation artificially produced in it by the introduction of a mineral acid sufficiently diluted with water to inject, without disorganizing, those portions of the tissues with which it comes in contact.

It is, besides, important to observe, that, *cœteris paribus*, the color of the gastro-intestinal mucous membrane presents some shades, according to, 1. the part examined; 2. the age; and 3. whether the process of digestion was going on or not in the stomach or in the duodenum and jejunum at the moment of death. Thus, in those cases in which the mucous membrane is found colorless in the adult, we may observe, as M. Billard has shewn us, that it is whitish in the stomach, of an ashy white in the duodenum and jejunum, that the ashy shade diminishes towards the end of the ileum, and that, finally, in the great intestine, the mucous membrane resumes its dead white color. With respect to age, we learn from the valuable researches of M. Billard, that the gastro-intestinal mucous membrane is rose-colored in the fœtus and in the infant, and of a milky and satiny whiteness in young persons; that, in the adult, it assumes a slight ashy shade, especially in the duodenum and commencement of the small intestine; and lastly, that in old age this ashy shade becomes more decided and general, whilst the submucous veins, being dilated and filled with blood, lift up and impart a color to the membrane covering them. At other times, however, in old persons who die in a decrepid and bloodless state, the mucous membrane is remarkable for its extreme paleness. I am even persuaded that it is in old persons, and in very young children that had died of marasmus, that I have observed the internal surface of the alimentary canal in the most perfectly colorless state.

I have already spoken of the modifications produced in the color of this membrane by the process of digestion.

The natural thickness and consistence of the mucous membrane of the alimentary canal are not less important to be determined precisely than its color.

This membrane, in its natural state, is far from being equally thick throughout. M. Billard has proved, that the *maximum* of the thickness exists in the duodenum, and the *minimum* in the colon. Between these two extremities we find, 1. the pyloric portion of the stomach, in which the mucous membrane of the stomach is almost as thick as the duodenum; 2. its splenic portion, where its thickness is much less; 3. the rectum; 4. the jejunum; 5. the ileum. The great thickness of the mucous membrane of the duodenum depends principally on the numerous folicles distributed through it; in the stomach, it is the body of the membrane itself which has an excess of thickness. M. Louis has attempted to measure exactly the relative thickness of the different portions of the mucous membrane of the stomach. Its thickness in the great curvature amounts, according to him, to three-fourths of a *millimetre;* in the small curvature from a third to three-fourths; and the great extremity, from a third to three-fifths only. (A *millimetre* is .03937 of an English inch.) There are certain folds formed by the mucous membrane, both in the stomach and elsewhere, which contribute to an apparent augmentation of its thickness. Where these folds exist, it often happens that there is a more extensive redness found than in their intervals; this is observed, for instance, in the *valvulæ conniventes* of the small intestine. But when we separate the two reflected portions of membrane that constitute each valve, by drawing asunder their base at each side, the unfolded membrane does not appear any redder than the neighboring parts.

In the remarks I have just made on the thickness of the mucous membrane of the alimentary canal, I have supposed it to be examined in the body of a person that had died of an acute disease, and whose intestines were not gorged with too great a quantity of blood from one of the causes already pointed out. When that occurs, the thickness of the membrane may be increased by the blood which distends its vessels, without our being warranted in considering it as diseased on that account. On the other hand, in persons who die of marasmus, without having been affected with gastro-intestinal irritation, the mucous membrane of the alimentary canal becomes remarkably thin, falling into a state of atrophy along with the subjacent muscular coat: many other tissues are similarly affected under the same circumstances. This attenuation is particularly remarkable in the stomach, whose mucous membrane, especially towards the great extremity, becomes reduced to an exceedingly fine kind of web. I grant that a membrane so attenuated as that, is no longer in its physiological state, and that its functions must be deranged; it must digest but imperfectly, just as its muscular coat, under the same circumstances, must contract but imperfectly. At this degree, the attenuation of the parietes of the stomach becomes a morbid state; but before it reaches that state we may observe many other degrees in which the diminution of thickness of the mucous membrane of the stomach is physiologically proportionate to certain states of the general nutritive action. Lastly, I think it probable enough that great varieties of thickness of this mucous membrane must occur in individuals, just as is the case with the cutaneous system, and as there are also differences of bulk in the muscular and osseous systems in various individuals.

The consistence of the gastro-intestinal mucous membrane is in general directly in proportion to its thickness. It is much more considerable in the pyloric portion of the stomach than in its splenic portion; in the colon, where the thickness of the mucous membrane is at its *minimum*, its consistence is also very slight. In the stomach, we may allow the mucous membrane to be of the natural thickness, when, on making an incision in it, taking care not to cut the subjacent tissues, especially the nervous, or, more properly, the membranous coat, we can easily detach pretty considerable shreds of it with a forceps: the shreds should be larger in the pyloric than in the splenic portion. In the duodenum its nature is such as not to admit of such considerable shreds being detached as in the stomach. In the rest of the intestines, the rectum excepted, the mucous membrane, even in its natural state, breaks and tears whenever we attempt to detach any portion of it. In these various parts, however, the same physiological conditions which produce a variation in the thickness of the membrane, such as the quantity of blood supplying it, and the general state of the nutritive powers, produce a variation in its consistence. Thus, at the same time that this membrane becomes thinner, it tends also to grow softer, without the previous or present existence of any process of irritation.

The mucous membrane of the alimentary canal may, after death, be modified in its consistence, as we have already seen it to be in its color. This kind of softening has been observed principally in two cases: 1. long after death, when there were already signs of putrefaction in the body; 2. in a very short period after death.

In the first of these cases the membrane loses its consistence but slowly. I have more than once found it not in the slightest degree softened in bodies of persons that had been from eight to ten days dead, in which the intestines were green and distended with gases, while there was exudation of blood into them, together with ecchymoses in the substance of their parietes, and in many parts, emphysema under the membrane. After the tenth day its consistence diminishes, and it then softens gradually; from the fifteenth to the eighteenth day it becomes like pap, and from the twenty-fifth to the thirtieth it becomes quite undistinguishable.

This membrane, when exposed to the air, softens much more rapidly. M. Billard, after opening an intestinal canal, left it extended on a table for twelve days; the temperature of the room was ten degrees above zero, and the sun shown into it every day. The mucous membrane did not begin to soften until the sixth day, at which period putrefaction was already advanced; on the tenth day it was a pultaceous consistence; and, on the eleventh, it was reduced to a very fetid greenish pulp.

On the contrary, this membrane, when removed from the influence of the atmosphere by being placed under water, softens but very slowly. M. Billard, after leaving a portion of intestine for two months in the same water, and not till then, found its mucous membrane perceptibly softened, though it still retained a certain degree of consistence. It was not till three months had elapsed, that it was found to be so softened as to resemble merely a kind of very fetid purulent layer.

It follows from these facts that the *post-mortem* softening of the gastro-intestinal mucous membrane does not occur until the putrefaction is pretty far advanced, and after the usual period of opening bodies in most cases. It would appear, then, that we should not consider the very evident softening of the mucous membrane of the stomach, that is sometimes observed at from twenty to four and twenty hours after death, to have taken place after that event. However, the solution of this question is embarrassed, if I may say so, by some cases in which the mucous membrane of the stomach has been found completely softened, in dogs killed in very good health, and opened shortly after death. Similar facts have been observed by M. Bretonneau. M. Trosseau, who gives an account of them in the *Archives de Medecine* (tom. xii. p. 345) adopts an opinion of Hunter's, who has numerous followers in England at the present day, and attributes this kind of softening to the solvent action of the juices secreted by the stomach. According to several English physicians, the softening might even extend to all the coats of the stomach, and produce a perforation of that viscus after death. The arguments on which they ground their opinion are the following.

1. It has frequently occurred, that on opening the bodies of men, and still more of various animals, the parietes of the stomach have been found singularly softened or perforated, although before death there had been no symptoms of any gastric affection, and most of them had been killed while apparently in the enjoyment of perfect health. Adams has found such perforations in the stomachs of dogs; Carlisle and Cooper in those of rabbits; and Spallanzani in those of fishes. Before that, Hunter had found the stomach perforated in a prisoner who had starved himself to death.

2. In these cases, no traces of peritonitis were discovered around the perforation; which should necessarily have occurred if the communication between the cavity of the stomach and that of the peritoneum had been formed during life.

3. Doctor Allan Burns found, in a human body, the stomach perforated in that part where it is in contact with the liver. Having ascertained that the latter was uninjured, he proceeded, two days afterwards to examine the parts anew, when he found that new changes had taken place; the portion of liver which supplied the place of the deficient parietes of the stomach was itself remarkably softened and broken down: still, there was as yet no sign of putrefaction in the body. In this case, as Mr. Burns observes, it is not evident that this klnd of liquefaction of the liver was owing to the solvent action of the same juice which had previously attacked and destroyed a portion of the gastric parictes?

4. From a young man of a sound constitution, who had a fistula of the stomach in the epigastrium, in consequence of a gunshot wound, Dr. Lovell, surgeon in chief of the armies of the United States, collected a certain quantity of juice which flowed out through the fistula. The juice, which was received in a bottle, was put in contact with some meat, when it was observed that it dissolved it with great activity from the surface to the centre: the meat, says the narrator of the experiment, dissolved like a piece of gum arabic when kept in the mouth. Now, if the gastric juice is so active as that, is it not easy to conceive that if, after death, it happens to be in the stomach in a certain quantity, and possessing certain qualities, it may dissolve the coats of that viscus, just as it dissolved the dead flesh in the experiment?

Are we then to admit, as proved by these facts, that the parietes of the stomach may be softened and perforated by the gastric juice after death? I think, myself, that the facts should be taken into consideration, but that they are neither sufficiently numerous, nor circumstantially enough detailed, for us not to wait for new observations on the subject to confirm or contradict the conclusion drawn from them.

As the follicles, both isolated and aggregated, that are distributed over the gastro-intestinal mucous membrane, play a principal part in its diseases, it is of importance to determine accurately what varieties of appearance they may present, without yet ceasing to be in their physiological state.

In most adult subjects in which the alimentary canal does not appear to have undergone any alteration, very manifest follicles are found but in two parts; namely, around the cardiac orifice of the stomach, and in the duodenum. Moreover, in the inferior portion of the ileum, there are certain spots found where the parietes of the intestine seem to the touch to be thicker than elsewhere, and on being placed between the eye and the light, have not the usual degree of transparency. It is there that those aggravated follicles, known by the name of *the glands of Peyer* make their appearance in other subjects.

In children the follicles are naturally more developed, and appear in a greater number of parts. Thus, in them, even when there has been no indication of the existence of any intestinal affection, it is quite common to find on the internal surface both of the small and of the great intestine small round bodies, of a white or greyish color, and with a central orifice, the circumference of which is very often of a deep grey; which are, very evidently, nothing but follicles. In many children, too, in whom the intestinal canal appears equally free from any morbid alteration, besides these isolated follicles, others are found crowded together, and lying close on one another in immense numbers, so as to form by their assemblage, very large patches that may occupy an extent of from one to three feet in the small intestine. In the centre of each follicle is often found a point of bluish grey or black; which produces a dotted appearance throughout the patch. As I have found these patches (*the aggregated glands* of Peyer) thus far developed, and thus colored, in children who died suddenly in consequence of accidents, or of diseases that had no relation whatever to the digestive apparatus, I think I may assume as a fact, that they do not constitute a morbid state in childhood. But are they a proof of disease in the adult? It is true enough that, in many persons that die of or with chronic diarrhœa, the most remarkable and striking change found in the alimentary canal, is an unusual developement of the follicles, resembling what we have just now seen existing naturally in children. Besides, of many other individuals who died of some other disease while recovering from gastro-enteritis attended with severe symptoms, such as are termed adynamic and ataxic, I have often found on the internal surface of the alimentary canal, near the end of the ileum, the aggregated glands of Peyer appearing in the form of vast patches dotted with black. I believe that, in this latter case, the dotted patches discovered on examination after death, indicated that the follicles were in a state of hypertrophy, resulting from the recent irritation that had affected them. But this state of hypertrophy might have continued without producing any bad effect, as is proved by the fact that in many other adults the follicles are found in a similar state, although at the time of their death there had been no sort of disease in the *primæ viæ*. To conclude then, I think that the great developement of the intestinal follicles is not a natural state in the adult: it may result from an antecedent process of acute inflammation, and continue as a vestige of such process; it may also occur without any appreciable trace of antecedent irritation, being connected simply with an increased action of the regular process of nutrition; or, if you please, it may depend on the nutrition of these follicles continuing to be as active in the adult as it was in the child. It is thus that the liver may continue as much developed in the adult as it was in the early period of its existence; it is thus, too, that in a man of middle age, although free from disease, the different lymphatic ganglions may be found as large as they are in children.

It follows from these considerations that the intestinal follicles, as well those which are isolated as those which by their aggregation towards the inferior portion of the ileum, form the plexus of Peyer, may, in the adult be sometimes apparent, and sometimes almost imperceptible, without either of these states being really morbid; so far as that each may occur without producing any disorder of the digestive functions. We learn, besides, from comparative anatomy, that a very great developement of these follicles is natural in some animals. Thus, in most of the dogs that are sacrificed to physiological experiments, there are found in the small intestine numerous extensive patches dotted with black, exactly resembling those found also in the human subject, but yet so far from constantly, that when they are met with they are considered as morbid. They have been likewise observed in the intestinal canal of numbers of sheep killed in the slaughter houses. I have also very often discovered them in horses; but as I know not whether they had had any affection of the *primæ viæ* or not, I cannot, in their case, as in that of the other animals, bring forward the existence of these patches as a proof that they belonged to the healthy state.

The tissues subjacent to the mucous membrane present, in the healthy state, the following characters.

The submucous cellular tissue ought to have the appearance of a white layer, of a pretty great density, and traversed or not by a certain number of veins more or less gorged with blood. The muscular coat should be pale, resembling in color the muscles of white blooded animals; it should have its *maximum* of thickness in the pyloric portion of the stomach and in the rectum, and should appear thick in proportion to the contraction of the intestine. In persons who die in a state of marasmus, without any chronic irritation of the intestinal canal, the gastro-intestinal muscular coat becomes remarkably wasted. The cellular tissue interposed between this coat and the peritoneum is almost imperceptible in the healthy state: we must attend to this fact, because, in certain morbid states, this tissue may become hypertrophied, and contribute to the formation of various tumors. Nothing is more variable than the capacity of the intestines in different bodies. But we must take particular notice that we may find the intestinal tube exceedingly narrowed on the one hand, without its presenting any appreciable lesion.; and exceedingly wide, on the other, although presenting indubitable traces of irritation. Sometimes it is found narrowed in a single spot, so as to form a kind of strangulation; while, either above or below it, the intestine is very wide: there is, however, no more trace of irritation in the contracted part than elsewhere. I have frequently observed a great whiteness on the internal surface of stomachs that were so shrunk as hardly to surpass the bulk of the colon.

I have found that other stomachs, which had been disorganized by the action of sulphuric acid, were very large.

The internal surface of the alimentary canal ought to be lubricated, in the healthy state, by a moderate quantity of greyish viscous mucus, capable of being collected, in form of a pretty consistent pulp, on the blade of the scalpel, by slightly scraping the membrane with it. In the stomach, this mucus, when not mixed with the *ingesta*, is in a state of purity; it accumulates and is ultimately digested when no aliment has been taken for a long time. The secretion of this mucus is promoted by introducing some inert body into the stomach; such as a pebble, round which it collects, or a sponge, which may then be withdrawn impregnated with it. In the small intestine, it is mixed with a certain quantity of bile; in the great intestine its place is supplied by fæces, which are often found in great quantities in the colon of persons who yet have not eaten any thing for a long time."

